# Scoring systems for predicting clinical outcomes in peptic ulcer bleeding

**DOI:** 10.1097/MD.0000000000030410

**Published:** 2022-09-09

**Authors:** Jin Hee Noh, Boram Cha, Ji Yong Ahn, Hee Kyong Na, Jeong Hoon Lee, Kee Wook Jung, Do Hoon Kim, Kee Don Choi, Ho June Song, Gin Hyug Lee, Hwoon-Yong Jung

**Affiliations:** a Department of Gastroenterology, Asan Medical Center, University of Ulsan College of Medicine, Seoul, Korea; b Division of Gastroenterology, Department of Internal Medicine, Inha University School of Medicine, Incheon, Korea.

**Keywords:** AIMS65, Glasgow–Blatchford score, Peptic ulcer bleeding, Rockall score, upper gastrointestinal bleeding

## Abstract

Few studies have focused on assessing the usefulness of scoring systems such as the Rockall score (RS), Glasgow–Blatchford score (GBS), and AIMS65 score for risk stratification and prognosis prediction in peptic ulcer bleeding patients. This study aimed to assess scoring systems in predicting clinical outcomes of patients with peptic ulcer bleeding. A total of 682 peptic ulcer bleeding patients who underwent esophagogastroduodenoscopy between January 2013 and December 2017 were found eligible for this study. The area under the receiver-operating characteristic curve (AUROC) of each score was calculated for predicting rebleeding, hospitalization, blood transfusion, and mortality. The median age of patients was 64 (interquartile range, 56–75) years. Of the patients, 74.9% were men, and 373 underwent endoscopic intervention. The median RS, GBS, and AIMS65 scores were significantly higher in patients who underwent endoscopic intervention than in those who did not. The AUROC of RS for predicting rebleeding was significantly higher than that of GBS (*P* = .022) or AIMS65 (*P* < .001). GBS best predicted the need for blood transfusion than either pre-RS (*P* = .013) or AIMS65 (*P* = .001). AIMS65 score showed the highest AUROC for mortality (0.652 vs. 0.622 vs. 0.691). RS was significantly associated with rebleeding (odds ratio, 1.430; *P* < .001) and overall survival (hazard ratio, 1.217; *P* < .001). The RS, GBS, and AIMS65 scoring systems are acceptable tools for predicting clinical outcomes in peptic ulcer bleeding. RS is an independent prognostic factor of rebleeding and overall survival.

## 1. Introduction

Upper gastrointestinal (GI) bleeding is one of the most common emergency diseases of the digestive system, with high mortality and morbidity rates. In general, upper GI bleeding is classified into variceal bleeding and nonvariceal bleeding. The most common cause of nonvariceal bleeding is peptic ulcer, accounting for 25%–67%, followed by other causes such as malignancy, erosion, Mallory–Weiss syndrome, angiodysplasia, and esophagitis.^[1]^ As the mortality rate of upper GI bleeding is still high at 6%–14%, it is important to predict the clinical course and prognosis of patients, classify high-risk groups, and establish appropriate treatment plans according to risk stratification.^[2,3]^

Several scoring systems for assessing the risk of patients with upper GI bleeding have been validated, including the widely used Rockall score (RS), Glasgow–Blatchford score (GBS), and AIMS65 score. Although the role of scoring systems in clinical practice remains uncertain, there have been several reports on the usefulness of scoring systems for risk stratification and prognosis prediction in patients with upper GI bleeding.^[4–6]^ Most of the studies have included patients with nonvariceal and/or variceal upper GI bleeding. However, few studies have focused on peptic ulcer bleeding, which is the most common cause of upper GI bleeding.^[7]^

Therefore, in this study, we aimed to investigate patients who visited the emergency department with peptic ulcer bleeding and to compare the clinical outcomes and median scores between patients who underwent endoscopic intervention and those who did not. Further, we aimed to assess the RS, GBS, and AIMS65 scoring systems for predicting rebleeding, hospitalization, blood transfusion, and mortality.

## 2. Materials and Methods

### 2.1. Patients

Between January 2013 and December 2017, a total of 5076 patients underwent esophagogastroduodenoscopy (EGD) for suspected upper GI bleeding at Asan Medical Center, a tertiary university hospital in Seoul, Korea. Of them, 4212 patients were excluded for the following reasons: variceal bleeding (n = 1154); cancer bleeding (n = 538); lower GI bleeding (n = 381); postprocedural bleeding (n = 300); other causes of GI bleeding (n = 1839), including esophagitis, Mallory–Weiss tear, gastric erosion, vascular ectasia, acute gastroduodenal mucosal lesion, feeding tube injury, and hemobilia. Of the remaining 864 patients with peptic ulcer bleeding, 182 patients who had a bleeding event during hospitalization were excluded, since there may be other factors associated with hospitalization that can affect and increase the score, such as comorbidity, underlying disease, hypoalbuminemia, etc. Finally, a total of 682 patients who visited the emergency department and underwent EGD for peptic ulcer bleeding were enrolled in this study. The patients were divided into two groups based on their history of endoscopic intervention for hemostasis: one group with endoscopic intervention (n = 373) and the other without endoscopic intervention (n = 309).

The clinical features and median scores of endoscopic factors were retrospectively investigated and compared between the groups with and without endoscopic intervention. Informed consent for performing EGD was obtained from all patients before the procedure. The institutional review board of Asan Medical Center approved this study (approval no. 2020-0106).

### 2.2. Management and definitions

Upper GI bleeding was defined as chief complaints of hematemesis, melena, hematochezia with vital sign instability, and others including syncope and anemia.^[5]^ Patients suspected of having upper GI bleeding received high-dose acid suppression therapy (esomeprazole or pantoprazole 80 mg intravenous bolus, followed by continuous infusion at 8 mL/h for 72 h) according to the judgment of physicians and depending on the patient’s clinical status. Posthemorrhagic anemia was corrected with red blood cell transfusion if the hemoglobin level was below 7 g/dL. However, for a large amount of acute bleeding with hemodynamic instability, transfusion was performed regardless of the results of blood tests.

Peptic ulcer bleeding was stratified according to endoscopic appearance using the Forrest classification (Ia, spurting bleeding; Ib, oozing bleeding; IIa, nonbleeding visible vessel; IIb, adherent clot; IIc, hematin on ulcer base; III, clean ulcer base). Endoscopic hemostasis was performed under the judgment of the attending physician who initially performed the endoscopy. Further, embolization was considered in active bleeding cases with hemodynamic instability or if endoscopic intervention failed and massive bleeding persisted in the patient.

Rebleeding was defined as one or more signs (among hematemesis, melena, hematochezia, bloody drainage from the nasogastric tube, hemodynamic instability, and > 2 g/dL reduction in hemoglobin levels) occurring within 7 days after the primary endoscopic intervention and a need for additional endoscopic intervention. The duration of hospitalization was defined as the interval from the day of the emergency department visit to the day of discharge. The follow-up period was defined as the interval from the endoscopy day to the last outpatient clinic visit. The survival status of patients was obtained from their medical records and National Health Insurance Service records (South Korea).

### 2.3. Evaluation of scoring systems

The collected data were used for calculating the RS, GBS, and AIMS65 scores. The post-endoscopic clinical RS which is the most commonly used scoring system including age, signs of shock, comorbidities, endoscopic diagnosis, and evidence of bleeding was investigated in this study. The GBS includes the following parameters: blood urea nitrogen, hemoglobin, systolic blood pressure, pulse rate, presence of melena or syncope, hepatic disease, and heart failure. It can be calculated simply, as it does not require endoscopic findings or the determination of the degree of systemic disease. The AIMS65 score is a more simplified scoring system and it consists of five risk factors: hypoalbuminemia, age > 65 years, low systolic blood pressure, altered mental status, and prolonged prothrombin time. The area under the receiver-operating characteristic curve (AUROC) of each scoring system was calculated and compared for predicting rebleeding, hospitalization, blood transfusion, and mortality.

### 2.4. Statistical analysis

Descriptive variables are summarized as median (interquartile range [IQR]). Differences in patient characteristics between the two groups were appropriately compared using independent t-tests and the chi-square test. Cox regression analysis was performed to determine the significant factors affecting overall survival. The ability of the scoring systems to predict outcomes was evaluated using AUROCs with 95% confidence intervals (CIs). The AUROC of each scoring system was compared using the DeLong method, and Youden’s index was utilized to determine optimal cut points. A value of *P* < .05 was considered to indicate statistical significance. All statistical analyses were performed using SPSS version 24 (IBM Corporation, Somers, New York) and MedCalc (version 19.6.3; MedCalc Software, Ostend, Belgium).

## 3. Results

Table 1 shows the clinical characteristics and endoscopic outcomes of patients with peptic ulcer bleeding with or without endoscopic intervention. The median age was 64 years (IQR, 56–75 years), and 74.9% of the patients were men. Of the patients, 43.2% had a smoking history and 46.7% habitually consumed alcohol. Moreover, 26.1% of the patients had a medication history including the use of antiplatelet agents and anticoagulants. The 30-day mortality rate was 4.3%, and the median duration of hospitalization was 3 days (IQR, 1–6 days). A total of 373 patients underwent endoscopic intervention. The median follow-up period was 52.0 months (IQR, 34–70 months).

**Table 1:** Clinical characteristics and endoscopic outcomes in patients with peptic ulcer bleeding with or without endoscopic intervention.

	With endoscopic intervention (n = 373)	Without endoscopic intervention (n = 309)	*P* value
Age at diagnosis, years (IQR)	63 (55–74)	66 (57–75)	.032
Male sex, n (%)	284 (76.1)	227 (73.5)	.422
Alcohol			.003
Nonuser	177 (47.5)	187 (60.5)	
Ex user	62 (16.6)	38 (12.3)	
Current user	134 (35.9)	84 (27.2)	
Smoking			<.001
Nonuser	184 (49.3)	203 (65.7)	
Ex user	91 (24.4)	49 (15.9)	
Current user	98 (26.3)	57 (18.4)	
Chief complaint			
Hematemesis	123 (33.0)	55 (17.8)	<.001
Melena	183 (49.1)	165 (53.4)	.259
Hematochezia	36 (9.7)	34 (11.0)	.563
Syncope	9 (2.4)	12 (3.9)	.268
Anemia	22 (5.9)	43 (13.9)	<.001
Underlying disease	313 (83.9)	271 (87.7)	.160
Antithrombotics	91 (24.4)	87 (28.2)	.266
Antiplatelet agents	65 (17.4)	66 (21.4)	
Anticoagulants	26 (7.0)	21 (6.8)	
Initial sBP < 100 mmHg	126 (33.8)	52 (16.8)	<.001
Initial pulse rate > 100/min	156 (41.8)	94 (30.4)	.002
Laboratory data			
Hemoglobin, g/dL	8.8 (6.9–10.6)	8.7 (6.6–10.6)	.781
BUN, mg/dL	38.0 (26.0–52.0)	31.0 (20.0–52.0)	.069
Albumin (< 3 g/dL)	215 (57.6)	126 (40.8)	<.001
INR	1.1 (1.0–1.3)	1.1 (1.0–1.2)	.754
Follow-up period, months	51.0 (36.0–68.0)	53.0 (30.0–74.0)	.547
Endoscopy finding			.105
Gastric ulcer	225 (60.3)	205 (66.3)	
Duodenal ulcer	148 (39.7)	104 (33.7)	
Transfusion, packed RBC (n = 676)	3.0 (± 3.3)	2.3 (± 2.6)	.001
Rockall score	6.3 (± 1.6)	4.4 (± 1.6)	<.001
GBS	11.3 (± 3.5)	10.3 (± 3.6)	<.001
AIMS65	1.4 (± 1.0)	1.2 (± 1.0)	.002

Values are provided as median (interquartile range) and n (%).

BUN = blood urea nitrogen, GBS = Glasgow–Blatchford score, INR = international normalized ratio, RBC = red blood cell, sBP = systolic blood pressure.

### 3.1. Clinical characteristics

The endoscopic intervention group included more current smokers and current drinkers, and patients in this group significantly more frequently had hematemesis as the chief complaint (33.0% vs. 17.8%, *P* < .001). With respect to initial vital signs, hypotension (33.8% vs. 16.8%, *P* < .001) and tachycardia (41.8% vs. 30.4%, *P* = .002) were more frequently observed in the endoscopic intervention group. The gastric–duodenal ulcer ratio was 1.72:1, and 36.7% of the gastric ulcers were located in the lower third of the stomach. The median RS (6.3 vs. 4.4, *P* < .001), GBS (11.3 vs. 10.3, *P* < .001), and AIMS65 scores (1.4 vs. 1.2, *P* = .002) were significantly higher in the endoscopic intervention group than in the without intervention group.

### 3.2. Endoscopic outcomes

A total of 373 patients underwent endoscopic intervention, as follows: hemoclipping in 138 patients (37.0%), forceps coagulation in 93 patients (24.9%), argon plasma coagulation in 27 patients (7.2), glue injection in 79 patients (21.2), epinephrine injection in 33 patients (8%), and treatment failure in 3 patients (0.8%). Three patients with failed endoscopic intervention for duodenal ulcer bleeding had spontaneous hemostasis without any further intervention. Of the 373 patients who underwent endoscopic intervention, 157 underwent endoscopic hemostasis using two or more methods. The rebleeding rate was not significantly different between patients who underwent hemostasis using two or more methods and those who underwent only one method of hemostasis.

Embolization was more frequently performed in the endoscopic intervention group (2.4% vs. 0.3%, *P* = .026). The rebleeding rate after the initial EGD (8.3% vs. 1.3%, *P* < .001) and the hospitalization duration (4.0 vs. 2.0 days, *P* = .021) were also higher in the endoscopic intervention group than in the without intervention group. The overall survival and 30-day mortality rates were not significantly different between the two groups.

### 3.3. Association between clinical outcomes and scoring systems

The median scores of each clinical and endoscopic factor in patients with peptic ulcer bleeding are summarized in Table 2. The median RS of Forrest classification Ia to IIb was > 6, and the median RS of Forrest IIc and III was 4. All patients with RS ≤ 2 had Forrest IIc and III peptic ulcers, and these patients did not undergo endoscopic intervention. Of the 99 patients with RS ≤ 3, 17 (17.2%) had Forrest Ia to IIb peptic ulcers and 16 (16.2%) underwent endoscopic intervention. The distribution of Forrest classification according to each scoring system in patients with peptic ulcer patients was also analyzed (Figure S1, Supplementary Digital Content, http://links.lww.com/MD/H196).

**Table 2:** Median scores of each clinical and endoscopic factor in patients with peptic ulcer bleeding with or without endoscopic intervention.

	Rockall score		GBS		AIMS65	
	With intervention	Without intervention	With intervention	Without intervention	With intervention	Without intervention
Total	6 (5–8)	4 (3–5)	12 (9–14)	11 (8–13)	1 (1–2)	1 (0–2)
Gastric ulcer	6 (5–7)	5 (4–5)	12 (10–14)	11 (8–13)	2 (1–2)	1 (1–2)
Duodenal ulcer	6 (5–8)	4 (3–5)	12 (8–14)	10 (8–13)	1 (0–2)	1 (0–2)
Forrest classification						
Ia	7 (5–8)	N/A	11 (8–14)	N/A	1 (1–2)	N/A
Ib	6 (5–7)	N/A	12 (9–14)	N/A	1 (1–2)	N/A
IIa	6 (5–8)	7 (5.5–7.5)	12 (10–14)	11 (10–14)	1 (1–2)	1 (0.5–2.5)
IIb	6.5 (5–8)	6 (5–7)	11 (8.5–13.5)	11 (8–13)	1.5 (1–2)	1 (1–2)
IIc	N/A	4 (3–5)	N/A	11 (8–13)	N/A	1 (0–2)
III	N/A	4 (3.5–5)	N/A	10 (8–13)	N/A	1 (0–2)
Location						
Upper third of stomach	6 (5–7)	5 (4–5)	11 (9–13)	12 (10–13)	1 (1–2)	1 (1–2)
Middle third of stomach	7 (6–8)	5 (3–6)	12 (10–14)	9 (7–11)	2 (1–2)	1 (0–2)
Lower third of stomach	7 (5–8)	4 (4–5)	12 (10–14)	11 (8–13)	2 (1–2)	1 (1–2)
Duodenal bulb	6 (5–8)	4 (3–5)	12 (8–14)	10 (8–13)	1 (0–2)	1 (0–2)
Duodenum 2nd portion	8 (7–8)	5 (4–5)	13 (10–15)	8 (5–11)	1 (1–3)	1 (1–1)

GBS = Glasgow–Blatchford score, N/A = not applicable.

**Figure 1.**
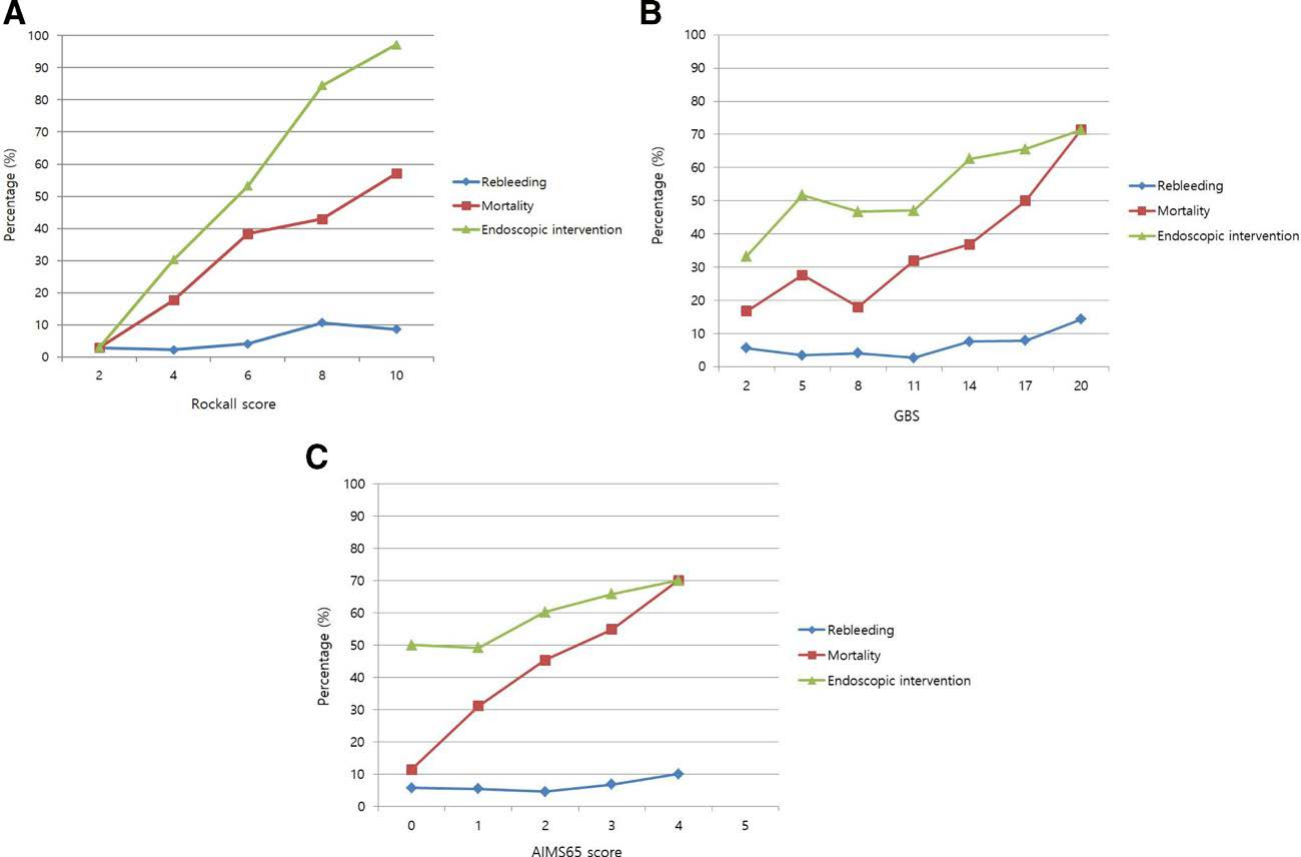
Rebleeding, mortality, and endoscopic intervention rates of patients with peptic ulcer bleeding. (A) Rockall score; (B) Glasgow–Blatchford score (GBS); (C) AIMS65 score.

Figure 1 shows the rebleeding, mortality, and endoscopic intervention rates of patients with peptic ulcer bleeding according to each scoring system. In all scoring systems, the percentage of each outcome tended to be higher as the scores became higher. In particular, no patient with RS 1 or 2 required endoscopic intervention.

### 3.4. Comparison of the prognostic value of the scoring systems

**Figure 2.**
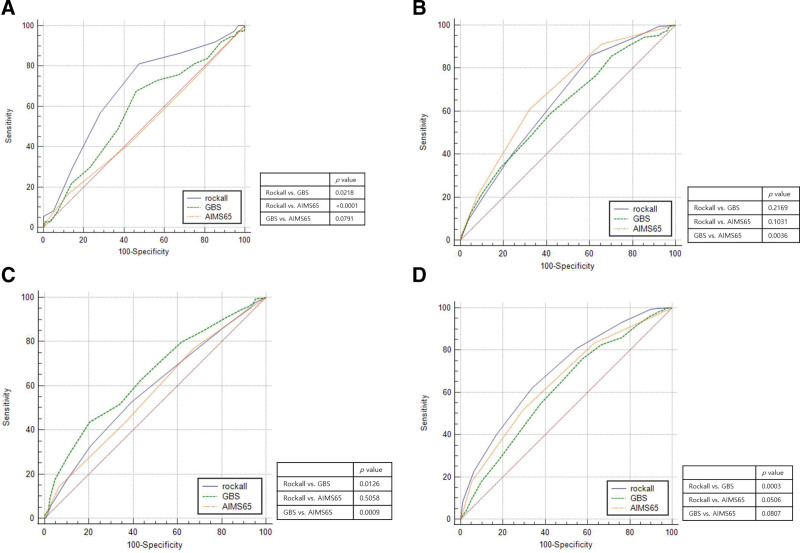
Comparison of areas under the receiver-operating characteristic curve (AUROCs) of the Rockall score, GBS, and AIMS65 score. (A) Rebleeding, AUROC 0.682 (95% CI, 0.645–0.716) vs. 0.583 (95% CI, 0.545–0.620) vs. 0.502 (95% CI, 0.464–0.540); (B) mortality, AUROC 0.652 (95% CI, 0.615–0.688) vs. 0.622 (95% CI, 0.584–0.659) vs. 0.691 (95% CI, 0.654–0.725); (C) need for transfusion, AUROC 0.583 (95% CI, 0.545–0.620) vs. 0.646 (95% CI, 0.609–0.682) vs. 0.566 (95% CI, 0.528–0.604); (D) hospitalization > 3 days, AUROC 0.697 (95% CI, 0.661–0.731) vs. 0.615 (95% CI, 0.577–0.652) vs. 0.654 (95% CI, 0.617–0.690).

The comparison of the AUROCs of RS, GBS, and AIMS65 scores for the prediction of clinical outcomes is shown in Figure 2. The AUROC of RS for predicting rebleeding was significantly higher than that of either GBS (0.682 vs. 0.583, *P* = .022) or AIMS65 score (0.682 vs. 0.502, *P* < .001; Fig. 2A). The sensitivity and specificity of RS were 81.1% and 52.6%, respectively, when the cut-off value was set at 5.5. The AIMS65 score showed the highest AUROC for mortality, with cut-off value 1.5 leading to maximal sensitivity (60.8%) and specificity (67.9%); it was superior to that of GBS (0.691 vs. 0.622, *P* = .004) and similar to that of RS (0.691 vs. 0.652, *P* = .103; Fig. 2B). According to the AUROC of each score, GBS for predicting the need for transfusion was significantly higher than that of either RS (0.646 vs. 0.583, *P* = .013) or AIMS65 score (0.646 vs. 0.566, *P* = .001; Fig. 2C). When the cut-off value was set at 12.5, the sensitivity and specificity of GBS were 43.4% and 79.8%, respectively. The RS showed the highest AUROC for prolonged hospitalization, which was superior to that of GBS (0.697 vs. 0.615, *P* = .000) and similar to that of AIMS65 (0.697 vs. 0.654, *P* = .051; Fig. 2D).

### 3.5. Risk factors for overall survival and rebleeding

Table 3 shows the independent risk factors for overall survival in patients with peptic ulcer bleeding. Liver cirrhosis (hazard ratio [HR], 2.226; 95% CI, 1.621–3.057; *P* < .001) was associated with poor survival. The RS (HR, 1.217; 95% CI, 1.107–1.338; *P* < .001) and AIMS65 scores (HR, 1.701; 95% CI, 1.471–1.966; *P* < .001) were significantly associated with overall survival. Further, multivariate analysis revealed that RS (odds ratio, 1.430; 95% CI, 1.177–1.736; *P* < .001) was an independent risk factor of rebleeding (Table S1, Supplementary Digital Content, http://links.lww.com/MD/H197).

**Table 3:** Cox proportional hazard model of factors associated with overall survival (n = 682).

	Univariate analysis		Multivariate analysis*	
	HR (95% CI)	*P* value	HR (95% CI)	*P* value
Sex, male	0.771 (0.579–1.025)	.074		
Liver cirrhosis	2.638 (1.941–3.584)	<.001	2.226 (1.621–3.057)	<.001
Chronic kidney disease	1.580 (1.174–2.127)	.003		
Antithrombotics	1.039 (0.775–1.394)	.798		
Initial sBP < 100 mmHg	1.379 (1.040–1.829)	.026	0.584 (0.411–0.831)	.002
Initial pulse rate > 100/min	1.008 (0.769–1.322)	.954		
Hemoglobin (< 8 g/dL)	1.687 (1.300–2.189)	<.001		
Albumin (< 3 g/dL)	2.842 (2.145–3.767)	<.001		
BUN, mg/dL	1.004 (0.999–1.008)	.099		
INR	1.269 (1.139–1.413)	<.001		
Rockall score	1.315 (1.222–1.415)	<.001	1.217 (1.107–1.338)	<.001
GBS	1.110 (1.067–1.155)	<.001		
AIMS65	1.759 (1.550–1.995)	<.001	1.701 (1.471–1.966)	<.001

BUN = blood urea nitrogen, CI = confidence interval, GBS = Glasgow–Blatchford score, HR = Hazard ratio, INR = international normalized ratio, sBP = systolic blood pressure.

*Simultaneously, adjusted for liver cirrhosis, chronic kidney disease, initial sBP < 100 mmHg, hemoglobin, albumin, INR, Rockall score, GBS, and AIMS65.

## 4. Discussion

The scoring systems used for predicting clinical outcomes in peptic ulcer bleeding patients who underwent EGD were investigated in this study. This study confirmed that the RS accurately predicts rebleeding after endoscopic intervention, and none of the patients with RS of 1 or 2 underwent endoscopic intervention for hemostasis. In addition, GBS was superior to RS and AIMS65 scores for predicting packed red blood cell transfusion. The AIMS65 score was superior to GBS and similar to RS in terms of predicting mortality. In predicting hospitalization, RS was superior to GBS and similar to the AIMS65 score.

Many studies have validated the usefulness of risk scoring systems in overall upper GI bleeding, whereas few studies have compared the risk scores specifically in peptic ulcer bleeding. Although the incidence of peptic ulcer has declined owing to *Helicobacter pylori* eradication and increasing use of proton pump inhibitors (PPIs), it remains an important disease entity as the use of antiplatelet drugs and nonsteroidal anti-inflammatory drugs is increasing owing to population aging and increasing incidence of arteriosclerotic and degenerative diseases.^[8,9]^ Therefore, we analyzed the predictive value of scoring systems only for peptic ulcer bleeding. All of the enrolled patients in our study underwent EGD and confirmed the bleeding focus as peptic ulcer.

Several studies have shown that AIMS65 was superior to GBS in predicting mortality during hospitalization.^[10,11]^ Similarly in our study, AIMS65 showed the highest AUROC in predicting mortality, and it was superior to GBS. Further, a recent study reported that AIMS65 was superior to RS in predicting mortality in patients with nonvariceal upper GI bleeding.^[12]^ However, in our study, which was limited to peptic ulcer bleeding, AIMS65 and RS showed no significant difference in predicting mortality, and both the scoring systems were significantly associated with overall survival. In predicting the need for blood transfusion, a previous study reported that GBS is superior to RS, which is similar to the results of our study.^[13]^ It is considered likewise because GBS includes hemoglobin as a parameter. The RS generally presents a better prognostic predictive power than GBS or AIMS65, which is thought to be due to the inclusion of endoscopic findings in RS. In this study, which was focused on peptic ulcer bleeding, RS showed the best prediction for rebleeding and prolonged hospitalization and confirmed the independent risk factor for rebleeding and overall survival.

Although many studies have compared the usefulness of the scoring systems, the results were inconsistent. However, risk stratification using scoring systems can reduce mortality and adverse events by identifying high-risk groups and those patients who need appropriate endoscopic intervention, in addition to reducing hospital stay durations and medical costs by identifying low-risk groups. In general, if the GBS is 0, the need for endoscopic intervention is within 1% and hospitalization or emergency endoscopy is not required.^[13–16]^ In our study, patients with an RS of ≤ 2 did not need endoscopic intervention for hemostasis. The proportion of patients with GBS 0 and RS 0, which predicted early discharge without endoscopic intervention, was reported to be approximately 5%–22% and 15%, respectively.^[17]^ Despite many efforts to expand the range, as many as 18% of patients classified in the low-risk group had clinical interventions, rebleeding, and death.^[17]^ Therefore, no scoring system has yet been able to completely screen patients in the low-risk group, and, ultimately, a final judgment based on the physician’s experience is needed accordingly.

This study had some limitations. First, selection bias may be present owing to the single-center and retrospective nature of the study. Second, the judgment of endoscopic findings and the selection of endoscopic intervention are endoscopist dependent, which can influence the clinical outcomes such as Forrest classification, performing hemostasis, or rebleeding rate. However, this study showed favorable outcomes in a large number of patients during a long-term follow-up period. Further, our study was investigated only in patients with peptic ulcer bleeding and all of them were performed EGD, therefore we could accurately the predictive value of RS was able to accurately evaluated.

In conclusion, the RS, GBS, and AIMS65 scoring systems are all acceptable tools for predicting rebleeding, hospitalization, blood transfusion, and mortality in patients with peptic ulcer bleeding. The RS is an independent risk factor of rebleeding and overall survival, and patients with RS ≤ 2 do not require endoscopic intervention and could be managed in the outpatient clinic.

## Author contributions

Conception and design: Ji Yong Ahn, Jin Hee Noh. Analysis and interpretation of the data: Jin Hee Noh, Boram Cha, Ji Yong Ahn, Hee Kyong Na, Jeong Hoon Lee, Kee Wook Jung, Do Hoon Kim, Kee Don Choi, Ho June Song, Gin Hyug Lee, Hwoon-Yong Jung. Drafting of the article: Jin Hee Noh. Critical revision of the article for intellectual content: Ji Yong Ahn.

## Supplementary Material


