# Supplementary material for: Scoring systems for predicting clinical outcomes in peptic ulcer bleeding
Source: Medicine (Baltimore). 2022 Sep 9;101(36):e30410. doi: 10.1097/MD.0000000000030410 (PMC10980471; doi:10.1097/MD.0000000000030410)
Supplement: Supplementary file 1 [file medi-101-e30410-s001.pdf]

Supplementary figure 1. Distribution of Forrest classification (presence of high risk stigmata; Ia–IIb vs. IIc–III) in patients with peptic ulcer bleeding (n = 682). (A) Rockall score; (B) Glasgow–Blatchford score (GBS); (C) AIMS65 score.

(A)

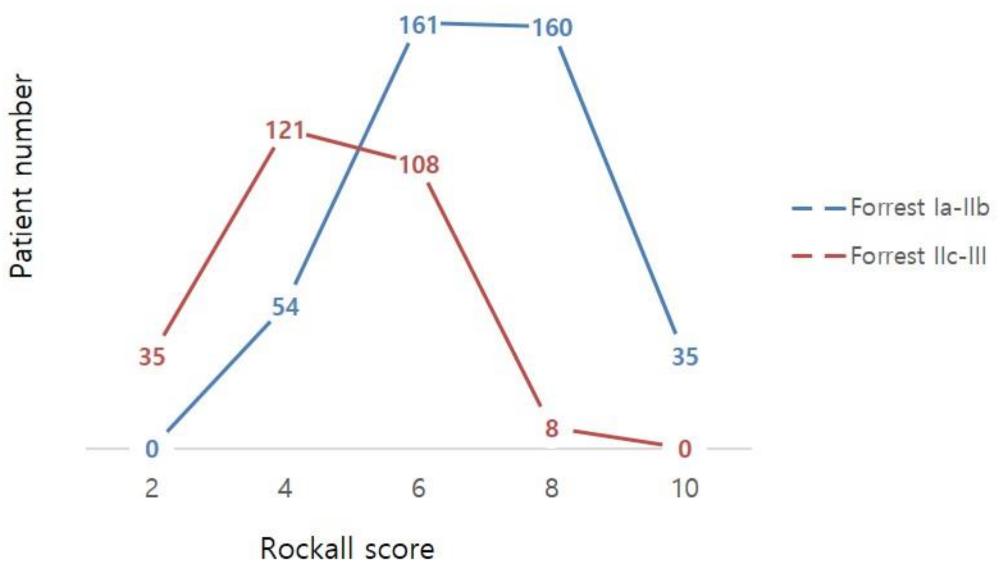

(B)

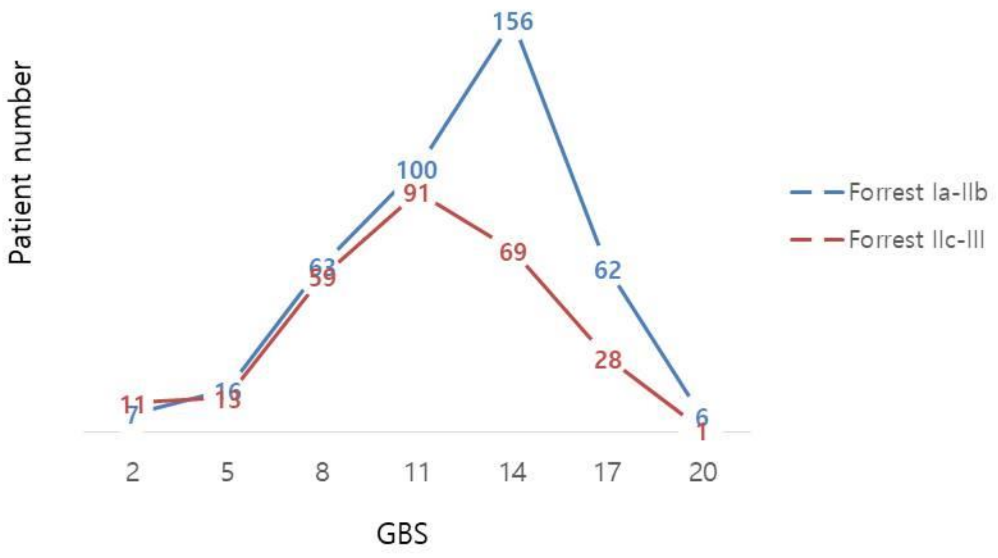

(C)

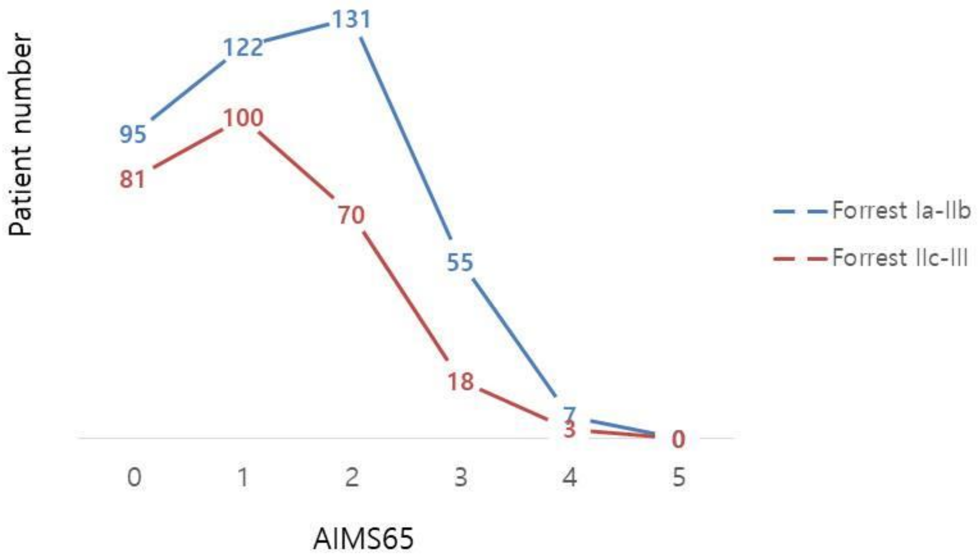
Patient number
131
122
95
100
81
70
55
18
7
3
0
0
1
2
3
4
5
AIMS65
Forrest Ia-IIb
Forrest IIc-III
