# Supplementary material for: Scoring systems for predicting clinical outcomes in peptic ulcer bleeding
Source: Medicine (Baltimore). 2022 Sep 9;101(36):e30410. doi: 10.1097/MD.0000000000030410 (PMC10980471; doi:10.1097/MD.0000000000030410)
Supplement: Supplementary file 2 [file medi-101-e30410-s002.pdf]

Supplementary table 1. Logistic regression analysis of factors associated with rebleeding in patients with peptic ulcer bleeding (n = 682)

	Rebleeding			
	Univariate analysis		Multivariate analysis	
	OR (95% CI)	*p*-Value	OR (95% CI)	*p*-Value
Sex, male	0.898 (0.425–1.896)	0.778		
Liver cirrhosis	1.180 (0.773–1.802)	0.443		
Chronic kidney disease	0.789 (0.322–1.933)	0.605		
Antithrombotics	0.647 (0.279–1.500)	0.310		
Initial sBP < 100 mmHg	1.785 (0.898–3.551)	0.099		
Initial pulse rate > 100/min	1.338 (0.685–2.615)	0.394		
Hb (< 8 g/dL)	1.136 (0.582–2.217)	0.710		
Albumin (< 3 g/dL)	1.333 (0.683–2.601)	0.399		
BUN, mg/dL	1.002 (0.990–1.014)	0.792		
INR	0.897 (0.549–1.467)	0.665		
Rockall score	1.430 (1.177–1.736)	< 0.001	1.430 (1.177–1.736)	< 0.001
GBS	1.075 (0.974–1.187)	0.149		
AIMS65	1.031 (0.744–1.428)	0.855		

BUN, blood urea nitrogen; CI, confidence interval; GBS, Glasgow–Blatchford score; Hb, hemoglobin; INR, international normalized ratio; sBP, systolic blood pressure
